# Insight in schizophrenia is associated with psychoeducation and social support: Testing a new more comprehensive insight tool in Turkish schizophrenia patients

**DOI:** 10.1371/journal.pone.0288177

**Published:** 2023-07-07

**Authors:** Ayse Gokcen Gundogmus, Philip Gerretsen, Jianmeng Song, Funda Erdi Akdag, Cagri Demirel, Ahmet Kokurcan, Sibel Orsel, Hasan Karadag, Kadir Ozdel

**Affiliations:** 1 Department of Psychiatry, Ankara Etlik City Hospital, Ankara, Turkey; 2 Multimodal Imaging Group, Research Imaging Centre, Centre for Addiction and Mental Health (CAMH), Toronto, Ontario, Canada; 3 Institute of Medical Science, University of Toronto, Toronto, Ontario, Canada; 4 Department of Psychiatry, Temerty Faculty of Medicine, University of Toronto, Toronto, Ontario, Canada; 5 Department of Psychiatry, Bergama Necla-Mithat Ozture State Hospital, Izmir, Turkey; 6 Department of Psychiatry, University of Health Sciences, Dr. Abdurrahman Yurtaslan Ankara Oncology Training and Research Hospital, Ankara, Turkey; 7 Department of Psychiatry, University of Health Sciences, Ankara Diskapi Yildirim Beyazit Training and Research Hospital, Ankara, Turkey; University of Catania Libraries and Documentation Centre: Universita degli Studi di Catania, ITALY

## Abstract

Insight is a continuous and multidimensional phenomenon, including awareness of having an illness, the presence of symptoms and accurate symptom attribution, the need for treatment, and the consequences of treatment. Good insight into illness is associated with better adherence to treatment, better cognitive, psychosocial, and vocational functioning along with less symptom severity, decreased relapses, and hospitalizations. Several tools are used for insight evaluation. We recruited 90 patients diagnosed with schizophrenia and analyzed the forms of 58 patients. The patients completed the VAGUS-SR (self-rated), Beck Cognitive Insight Scale, Knowledge About Schizophrenia Questionnaire, and Multidimensional Scale of Perceived Social Support (MSPSS). Clinicians performed a mental status examination and completed the Positive and Negative Syndrome Scale, Schedule for the Assessment of Insight, VAGUS-CR (clinician-rated), Calgary Depression Scale for Schizophrenia, and Clinical Global Impressions. We found that the level of insight evaluated using the VAGUS forms increased with knowledge regarding schizophrenia. Upon investigating the relationship between perceived social support and insight, we identified a relationship between VAGUS-CR and only significant other subscales of MSPSS, and between one of the VAGUS-SR scale sub-dimensions and significant other and total scores of MSPSS. Our findings also suggest that the VAGUS-SR and VAGUS-CR scales can be used to evaluate insight in Turkish populations. The positive relationship between perceived social support and insight emphasizes the importance of increasing social support through interventions aimed at improving insight. Our data also highlighted the value of psychoeducational studies in this patient group. Considering the multidimensional effects of insight on patients with schizophrenia, it would be beneficial to use scales such as VAGUS, which allow the insights of individuals to be evaluated in detail by both the clinician and the patient.

## Introduction

Studies on insight into schizophrenia have increased in recent years, and researchers have evaluated the concept of insight as a continuous and multidimensional phenomenon, including awareness of the illness, its symptoms and accurate symptom attribution, the need for treatment, and the consequences of treatment [1–6]. These dimensions are predominantly considered components of clinical insight [6]. However, insights into the disease can be evaluated in ways that are different from clinical insight [7, 8]. Clinical insight assessments are based on patients’ verbal reporting; therefore, they may occasionally indicate a repetition of previously heard information (intellectual insight). In contrast, cognitive insight is the ability of a patient to evaluate unusual experiences and recognize their misinterpretation, thereby integrating novel information into their thought processes and correcting their misconceptions [8, 9]. Metacognitive insight is the ability to monitor changes in one’s mental and sensory states and experiences [10]. Moreover, clinical, cognitive, and metacognitive insights may be associated with different neural correlates [4].

Although there is no consensus among clinicians regarding the definition and evaluation of insight, it has been reported that approximately 50–80% of individuals with schizophrenia experience a lack of insight in different dimensions [2]. The associations of insight, sociodemographic, clinical, and premorbid variables, and their effect on the course and outcome of the disease have been examined. However, insights are unstable, may be affected by current symptoms and previous characteristics (such as education level and premorbid functioning), and may change during disease progression [6, 11, 12]. Good insight into illness is associated with better adherence to treatment, better cognitive, psychosocial and vocational functioning, and less symptom severity, decreased relapses, fewer emergency room visits, hospitalizations, and hospitalization days [1–4, 6, 11, 13–16]. At the same time, good insight may be associated with lower self-esteem, poorer quality of life, and an increased risk of depression and suicide in psychotic disorders [3, 6, 11, 16–18]. Similarly, high metacognitive insight is associated with increased severity of depression [10].

Perceived social support and knowledge of schizophrenia were among the variables whose relationship with insight were investigated. A high level of knowledge regarding schizophrenia is associated with increased insight, but interestingly, psychoeducation does not exert a potent effect on improving insight [19–24]. The limited number of studies investigating the relationship between social support and insight in schizophrenia have conflicting results. Although some studies have described a curvilinear relationship between these features, others have not detected any relationship [14, 25, 26].

An important issue is the way insight is evaluated. The majority of scales that evaluate insight are clinician rated. However, clinician-rated instruments may run the risk of being overly affected by the clinician’s point of view, overlooking the beliefs and values of the patient, and are based on ratings of the individual’s observed behaviors and discourses rather than their personal experiences [27]. This feature underlines the importance of self-report scales, in addition to clinician-rated scales, for assessing insight because they provide information about a person’s views concerning the disease as well as their inner experiences [28]. The differences observed in the evaluations performed using self- and clinican-rated scales provide complementary rather than contradictory information about the individual’s insights [29]. Thus, we aimed to comprehensively evaluate insight in patients with schizophrenia by employing self-report and clinician-rated measures of insight and to assess the relationship between insight and particular clinical features. To accomplish this, we used the Turkish adaptation of the VAGUS insight into psychosis scale, which includes clinician-rated and self-report versions [30]. Subsequently, we aimed to investigate the relationship between insight and knowledge concerning schizophrenia, perceived social support, and psychotic and depressive symptom severity. We hypothesized that the VAGUS clinician-rated (VAGUS-CR) and VAGUS self-report (VAGUS-SR) versions are reliable and valid measurement tools for evaluating insight in Turkish patients with schizophrenia. Second, we expected that the level of knowledge concerning the disease and social support of the individual would be associated with greater insight and that the severity of depressive symptoms would increase with the level of insight.

## Methods

The study was performed at the University of Health Sciences, Ankara Diskapi Yildirim Beyazit Training and Research Hospital Psychiatry Clinic, and Community Mental Health Center (CMHC). Patients with schizophrenia were consecutively recruited to our study. The inclusion criteria were as follows: diagnosis of schizophrenia, age between 18 and 65 years, voluntary participation, and provision of informed consent. The exclusion criteria included psychiatric comorbidities, agitation or aggressive behavior, disorganized behavior, neurological disorders that can affect the cognitive status, a diagnosis of intellectual disability, an organic cause in the etiology of schizophrenia, an inability to fill in the self-report scales (Clinical Global Impressions, CGI > 4), and illiteracy.

All patients receiving treatment at abovementioned clinics were informed of the study protocol. We included those who agreed to participate and provided informed consent in accordance with the study criteria. First, the patients completed the VAGUS-SR, Beck Cognitive Insight Scale (BCIS), Knowledge About Schizophrenia Questionnaire (KASQ), and Multidimensional Scale of Perceived Social Support (MSPSS) tools. Subsequently, the clinicians performed a mental status examination, and the diagnosis of schizophrenia was confirmed with the Structured Clinical Interview for Diagnostic and Statistical Manual Disorders [31, 32]. The clinicians performed assessment using the Positive and Negative Syndrome Scale (PANSS), Schedule for the Assessment of Insight (SAI), VAGUS-CR, Calgary Depression Scale for Schizophrenia (CDSS), and CGI.

The study protocol was approved by the Ethics Committee of the University of Health Sciences, Ankara Diskapi Yildirim Beyazit Training and Research Hospital (03.02.2020-81/15). All the patients provided written informed consent, and the study was conducted in accordance with the Declaration of Helsinki.

### Measures

#### 1. Sociodemographic and clinical data questionnaire

This form was used to obtain information such as age, sex, marital and occupational status, education level, age of onset, and duration of disease.

#### 2. Positive and Negative Syndrome Scale (PANSS)

The PANSS was developed by Kay et al. [33] to assess positive, negative, and general symptom severity. It is a 30-item semi-structured interview scale that includes a seven-point assessment of symptom severity in schizophrenia. Seven items each belong to the positive and negative syndrome subscales, whereas sixteen items belong to the general psychopathology subscale. Researchers have previously determined the validity and reliability of the Turkish version of this scale [34]. In this scale, the score of item G12 (Lack of Judgment and Insight) is used to evaluate awareness of the disease and recognition of psychotic symptoms requiring treatment, with higher scores representing a more severe deficit in insight.

#### 3. Schedule for the Assessment of Insight (SAI)

David (1990) showed that insight cannot be evaluated as present or absent and developed the SAI that quantitatively evaluates insight based on three components: adherence to treatment, an awareness of the illness, and the recognition of psychotic experiences [35]. This semi-structured scale consists of eight questions evaluated by the clinician. Arlan et al. (2001) determined the reliability and validity of the Turkish version of the scale [36].

#### 4. VAGUS-CR and VAGUS-SR

Gerretsen et al. developed the VAGUS-CR and VAGUS-SR scale for easy application in psychotic disorders, increased sensitivity to marginal changes, and assessing different dimensions of insight [5, 27, 30]. Items in these scales are evaluated on a 10-point Likert scale.

VAGUS-CR consists of five items. General Illness Awareness, Awareness of Need for Treatment, and Awareness of Negative Consequences dimensions are evaluated using one item each. Two items of the Symptom Attribution dimension separately evaluate the attribution of delusions and auditory hallucinations to the disease. VAGUS-SR consists of 10 items and includes the following dimensions: “General Illness Awareness, Symptom Attribution, Awareness of the Need for Treatment, and Awareness of Negative Consequences.”

While adapting both versions of the VAGUS scale (VAGUS-CR and VAGUS-SR) to Turkish culture, we contacted the researchers who developed the measurement tool and obtained the necessary permission for its adaptation. Subsequently, the items on the scales, instructions, and scale scoring forms were translated into Turkish by three psychiatrists and revised by two of the authors (AGG and KO). Subsequently, the translations were disseminated to Gerretsen et al., who developed an original version of the scale for supervision and feedback. In line with the suggestions received, we finalized the Turkish version of the scale.

#### 5. Beck Cognitive Insight Scale (BCIS)

Beck et al. (2004) defined two sub-dimensions of cognitive insight, namely “self-reflectiveness” and “self-certainty,” and developed an insight scale to assess them [8]. In a previous study determining the reliability and validity of its Turkish version, researchers obtained Cronbach’s alpha of 0.56 and 0.50 for self-reflectiveness and self-certainty dimensions, respectively, in internal consistency measures [37]. In this study, Cronbach’s alpha values were 0.668 and 0.695 for self-reflectiveness and self-certainty, respectively. The items were rated by the participants on a four-point scale (“do not agree” to “agree completely”). While a high self-certainty subscale score indicates poorer cognitive insight, the self-reflectiveness subscale scores indicate the opposite. The composite index was calculated by subtracting self-certainty scores from self-reflectiveness, which reflected the patients’ cognitive insight adjusted for self-certainty.

#### 6. Clinical Global Impression—Severity of the Disease (CGI-S)

This is a seven-point Likert-type scale [38]. A clinician who is experienced with the disease and understands the rationale of scoring, rates the severity of the disease during the evaluation period. In this study, we obtained a Cronbach’s alpha of 0.712 for CGI-S, and its correlation with the total score of PANSS was 0.771 (p < 0.001). The items were scored between 1 and 7 according to the severity of the clinical condition: “1 = normal, 2 = borderline mental illness, 3 = mildly ill, 4 = moderately ill, 5 = significantly ill, 6 = severely ill, and 7 = among the most severely ill.”

#### 7. Calgary Depression Scale for Schizophrenia (CDSS)

This scale was developed by Addington et al. to assess depression, particularly in patients with schizophrenia [39]. The questionnaire was completed by the interviewer and consisted of nine items rated using a four-point Likert-type scale. Aydemir et al. (2000) determined the reliability and validity of the Turkish version [40]. Each item was scored between 0 and 3 points, and the total score was obtained by summing the individual scores.

#### 8. Knowledge About Schizophrenia Questionnaire (KASQ)

This self-report scale was developed by Ascher-Svanum [41]. It consists of 25 questions, including questions on the prevalence, etiology, course and prognosis, drug treatments and side effects, non-drug treatments, stressors, and legal processes, to assess awareness of schizophrenia and its management. The Turkish reliability and validity study was conducted by Atalan et al. [42], in which two questions were related to the legal rights of patients with schizophrenia. They were excluded from the evaluation because they did not comply with the laws of the Republic of Turkey, and the scores were eventually calculated for 23 questions.

#### 9. Multidimensional Scale of Perceived Social Support (MSPSS)

In 2001, Eker et al. performed the Turkish validity and reliability study of MSPSS, developed by Zimet et al. in 1988, which subjectively evaluates the adequacy of social support from three different sources [43, 44]. It consists of 12 items, and includes three groups, each consisting of four items regarding the source of support: family, friends, and significant others. Each item is rated using a seven-point scale. A higher score indicates greater perceived social support.

### Statistical analyses

Descriptive statistical measures (frequency and percentages), normality tests for the measurement tools (Kolmogorov–Smirnov and Shapiro–Wilk tests), and correlation analyses were performed. A confirmatory factor analysis (CFA) was performed to assess the construct validity of the adapted VAGUS scales. Moreover, we conducted CFA using the linear structural relations (LISREL) statistical package program (version 8.8). Cronbach’s alpha, stratified Cronbach’s alpha, and McDonald’s omega coefficients were calculated to determine the reliability. In addition, we used Spearman’s correlation coefficient to determine the convergent and discriminant validity of the VAGUS scales.

## Results

A total of 90 patients diagnosed with schizophrenia were enrolled. Of these patients, we excluded 22 patients who did not respond to either VAGUS-CR or VAGUS-SR, 8 who displayed extreme values (because of extreme value analysis), and 2 who could not be assigned missing data. The remaining 58 patients were analyzed. Table 1 summarizes the participants’ sociodemographic information.

**Table 1:** Clinical and demographic characteristics of patients.

		f	%
**Sex**	Female	19	32.8
	Male	39	67.2
**Education**	Literate	2	3.4
	Primary school	6	10.3
	Secondary school	10	17.2
	High school	25	43.1
	University	15	25.8
**History of psychoeducation**	Yes	32	55.2
	Min-max	$\overset{-}{X}$	S
**Age**	24–59	40.81	7.80
**Age of onset (years)**	15–34	22.45	4.65
**Duration of illness (years)**	2–33	16.91	7.62
**Numbers of admission**	0–10	3.14	2.61

Normality tests were performed and the distribution results of the measurement tools are provided as S1 Table. All scales, except the VAGUS-CR and CDSS, displayed a normal distribution.

### Data analysis

Before CFA, certain assumptions must be made to ensure consistent and accurate estimations. We first performed a missing data analysis because of missing data for particular items, which however did not exceed 5%. Therefore, we performed value assignment using the Expectation Maximization (EM) algorithm, which is a missing data value assignment method. The primary reason for using the EM algorithm is that it provides practical parameter estimations and unbiased results in case of fewer missing data [45]. Eight measurements were determined as extreme values. We calculated the multivariate skewness (Zc) and kurtosis (Zb) values, χ^2^ value for multivariate skewness and kurtosis, and relative multivariate kurtosis (RMK) values to assess the multivariate normal distribution assumption of the data set.

The VAGUS-CR form did not display a multivariate normal distribution, compared with the VAGUS-SR form (VAGUS-CR; Zc = 11.799 (p = 0.000), Zb = 5.165 (p = 0.000), χ^2^ = 165,899 (p = 0.000), and RMK = 1.669; VAGUS-SR, Zc = -0.103 (p = 0.918), Zb = 0.202 (p = 0.840), χ^2^ = 0.051 (p = 0.975), and RMK = 0.970). In the absence of multivariate normality, we preferred the robust maximum likelihood method as the parameter estimation method for the CFA. In contrast, we preferred the maximum likelihood method in the presence of multivariate normality. We examined bilateral correlations between the items and found no correlation > 0.80. In other words, there was no multicollinearity, and we observed linear relationships between the variables. At least five participants were required per item for sample size [46]. This assumption was met using the 10-item VAGUS-SR measurement tool.

### Validity of measurements obtained from the VAGUS-CR measurement tool

**Fig 1:**
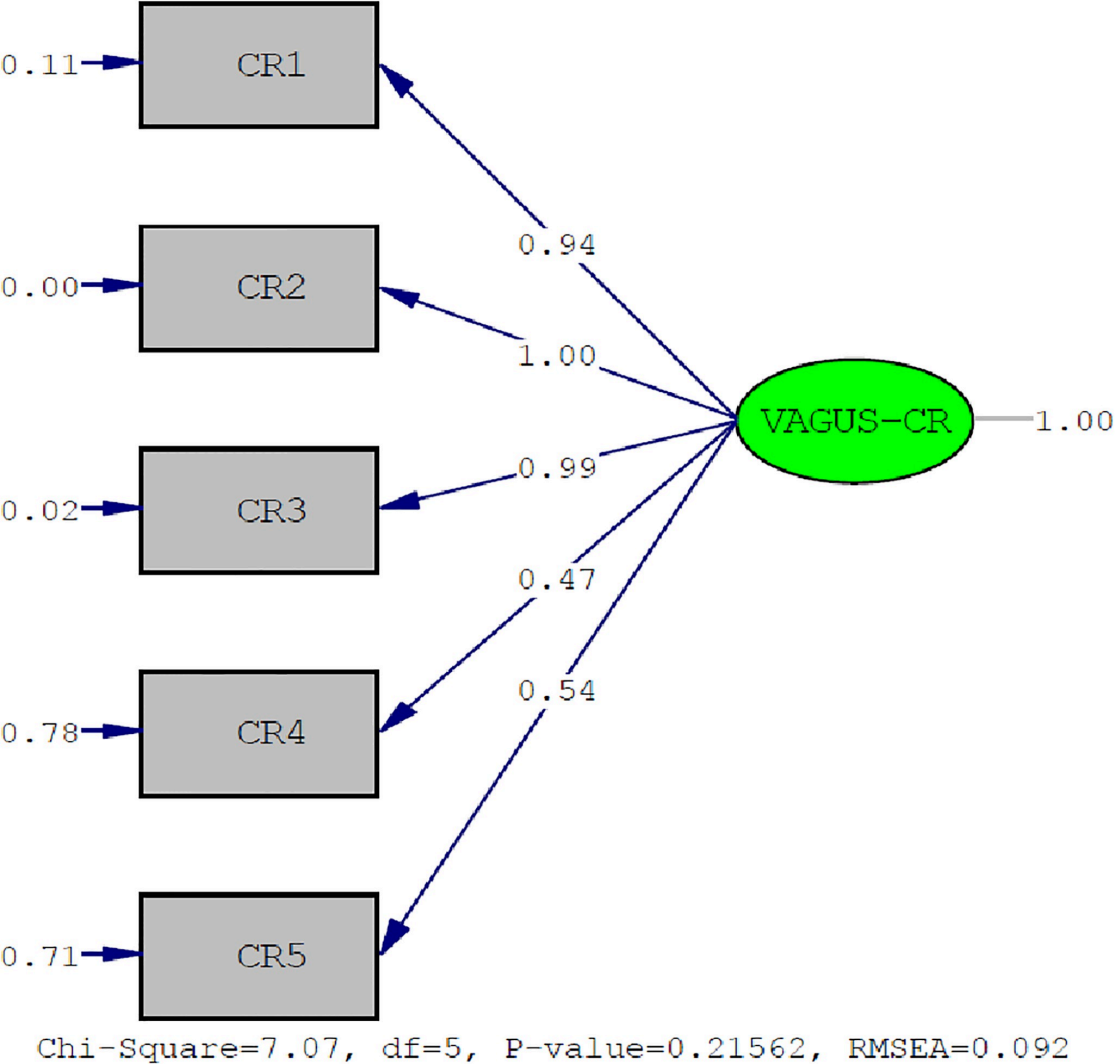
The measurement model obtained for the one-factor structure in the Turkish version of the VAGUS-CR (standardized path coefficients). VAGUS-CR, VAGUS clinician-rated.

Within the scope of this research, we performed CFA for conformity of the measurement model defined for the VAGUS-CR scale according to Turkish culture. The model-data fit values obtained from the analysis are provided in the S2 Table. Other fit indices were acceptable, except for the adjusted goodness of fit index (AGFI) and standardized root mean square residual values. Thus, we confirmed the single-factor structure of the measurement tool for Turkish culture. The data set did not meet the multivariate normality condition thereby generating low AGFI values. In such cases, we considered the comparative fit index and non-normed fit index instead of the goodness-of-fit index and AGFI [47]. Fig 1 shows the model with the fit values.

The factor loads (range, λ = 0.47–1.00) and error variance values (range, ε = 0.00–0.78) of the items in the measurement tool displayed acceptable values. Factor loads ≥ 0.30 indicated that the items were suitable for measuring the latent structure; error variances < 0.90 indicated an acceptable amount of error in the measurement of the latent structure [48]. After examining the general fit for CFA, we examined the modification indices and standardized residual values of the local fit of CFA. Eventually, there were no values ≥ 10 in the modification indices. Moreover, the standardized residual values were < 5%; therefore, a local fit of the model was achieved. Based on these findings, we confirmed the construct validity of the adapted VAGUS-CR measurement tool.

**Fig 2:**
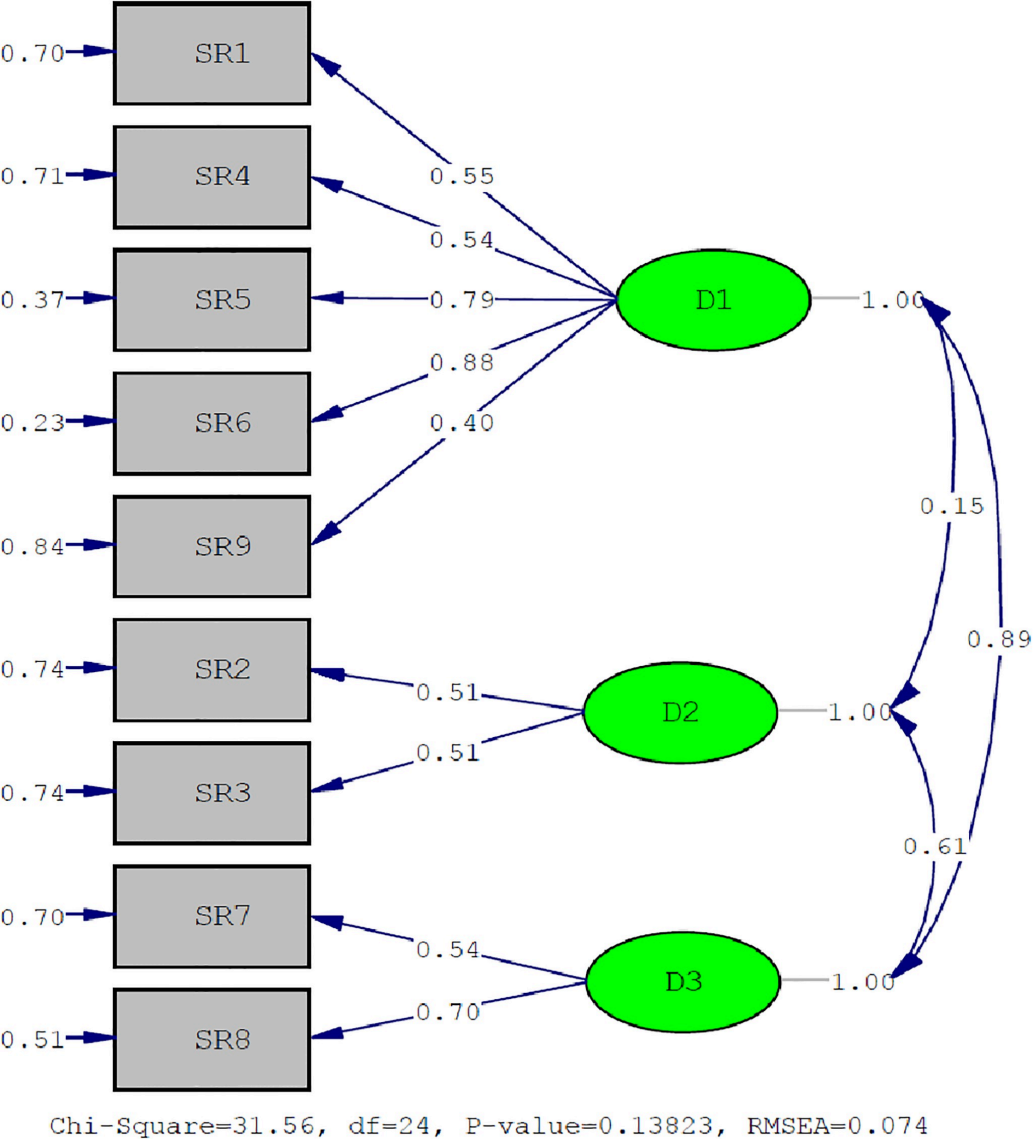
Measurement model regarding the factor structure of the Turkish version of the VAGUS-SR (standardized path coefficients). VAGUS-SR, VAGUS self-report.

A CFA was performed for the VAGUS-SR form, and the estimates obtained are provided in the S3 Table. The other fit indices were acceptable, except for the normed fit index, GFI, and AGFI values. Thus, we confirmed the three-factor structure of the measurement tool relevant to Turkish culture. Fig 2 shows the model with fit values.

The items in the measurement tool had factor loads (λ) ranging from 0.40 to 0.88; the error variance values (ε) ranged from 0.23 to 0.84. All the items had acceptable values. Factor loads ≥ 0.30 indicated that they were suitable for measuring the latent structure; error variances < 0.90 indicated an acceptable amount of error in the measurement of the latent structure [48]. Item SR10 was unsuitable for Turkish culture, and the factor load was almost zero (0.07); therefore, it was excluded from the study. We accepted the nine-item and three-factor structure of the VAGUS-SR form.

### Convergent and discriminant validity

After providing evidence of the construct validity of the VAGUS-CR and VAGUS-SR forms, Spearman’s correlation was used to provide evidence of convergent and discriminant validity. For the convergent validity of the VAGUS scales, we examined the pairwise correlations of VAGUS-CR with SAI and the G12 item of the PANSS scale, and VAGUS-SR with BCIS. For the discriminant validity of both the VAGUS-CR and VAGUS-SR scales, we examined the pairwise correlations of these scales with CDSS (Table 2). The VAGUS-CR form was negatively and strongly correlated with the G12 item of the PANSS scale, and positively and moderately correlated with SAI. There was no relationship between VAGUS-SR and CDSS. The CDSS was not related to SAI or the G12 item of PANSS. The VAGUS-SR scale displayed a significantly positive relationship with BCIS, whereas it had no relationship with CDSS.

**Table 2:** Convergent and discriminant validity analyses.

**VAGUS-CR**			
**Variables**	**VAGUS-CR**	**PANSS-G12**	**SAI**
VAGUS-CR	—		
PANSS-G12	-0.818*	—	
SAI	0.663*	-0.504*	—
CSDS	-0.012	0.014	0.004
**VAGUS-SR**			
**Variables**	**VAGUS-SR**	**BCIS**	
VAGUS-SR	—		
BCIS	0.346*	—	
CDSS	0.119	0.001	

*p ˂ 0.05

VAGUS-CR, VAGUS clinician-rated; VAGUS-SR, VAGUS self-report; SAI, Schedule for the Assessment of Insight; BCIS, Beck Cognitive Insight Scale; PANNS, Positive and Negative Syndrome Scale; and CDSS, Calgary Depression Scale for Schizophrenia.

### Internal consistency

We used the internal consistency coefficients, Cronbach’s alpha, stratified Cronbach’s alpha, and McDonald’s omega coefficients to provide evidence of the reliability of the VAGUS-SR and VAGUS-CR scales (Table 3). All scales and sub-dimensions, except for the second and third sub-dimensions of the VAGUS-SR form, displayed reliability values > 0.60 [49]. Despite low reliability of the second and third dimensions, a total score ≥ 0.70 indicated acceptable reliability. The primary reason for this was that similar participants responded to both the factors with low variance. The estimated reliability values of other measurement tools and descriptive statistics of all scales are provided in the S4 and S5 Tables.

**Table 3:** Estimated reliability values of VAGUS.

Scales and sub-dimensions	Cronbach α	McDonald ω	Stratified Cronbach α
VAGUS-CR	0.901	0.910	—
VAGUS-SR-D1	0.794	0.798	—
VAGUS-SR-D2	0.412	0.412	—
VAGUS-SR-D3	0.550	0.551	—
VAGUS-SR (Total)	—	—	0.834

VAGUS-CR, VAGUS clinician-rated; VAGUS-SR, VAGUS self-report

Furthermore, we examined the correlations between all the scales (Table 4). We observed a positive and moderately significant relationship between VAGUS-CR and VAGUS-SR. In addition, we identified a positive correlation between the sub-dimensions of VAGUS-SR. While the PANSS and its sub-dimensions were negatively associated with VAGUS-CR, they were not associated with VAGUS-SR. The G12 item of PANSS had a negative and moderate relationship with the VAGUS-CR and VAGUS-SR scales.

**Table 4:** Correlations between the VAGUS-SR and VAGUS-CR and other scales.

Scales and subscales	1	2	3	4	5
1. VAGUS-CR	—				
2. VAGUS-SR-D1	0.487**	—			
3. VAGUS-SR-D2	0.111	-0.016	—		
4. VAGUS-SR-D3	0.425**	0.472**	0.285*	—	
5. VAGUS-SR	0.500**	0.830**	0.429**	0.761**	—
SAI-total	0.663**	0.248	0.113	0.498**	0.361**
KASQ-total	0.412**	0.313*	0.033	0.343**	0.352**
MSPSS—Family	0.077	0.193	-0.050	0.032	0.135
MSPSS—Friends	0.124	0.195	-0.117	0.092	0.123
MSPSS—Significant other	0.277*	0.267*	0.111	0.098	0.258
MSPSS—Total	0.226	0.265*	-0.010	0.091	0.208
PANSS_positive	-0.300*	-0.139	0.166	0.183	0.039
PANSS_negative	-0.273*	-0.045	0.053	-0.081	-0.019
PANSS_general psychopathology	-0.427**	-0.153	0.012	-0.006	-0.087
PANNS_total	-0.438**	-0.125	0.057	-0.019	-0.052
PANNS_G12	-0.818**	-0.437**	-0.151	-0.224	-0.426**
CGI-S	-0.484**	-0.216	-0.042	0.016	-0.142
CDSS	-0.012	0.064	0.073	0.178	0.119
BCIS_self-reflectiveness	0.149	0.354**	0.043	0.052	0.307
BCIS_self-certainty	-0.092	0.036	-0.201	-0.232	-0.098
BCIS_composite_index	0.178	0.317	0.182	0.186	0.346**

*p˂.05;

**p˂.01.

SAI, Schedule for the Assessment of Insight; KASQ, Knowledge About Schizophrenia Questionnaire; BCIS, Beck Cognitive Insight Scale; MSPSS, Multidimensional Scale of Perceived Social Support; PANNS, Positive and Negative Syndrome Scale; CDSS, Calgary Depression Scale for Schizophrenia; and CGI-S, Clinical Global Impressions—Severity of the disease.

## Discussion

Our findings suggest that the VAGUS-SR and VAGUS-CR scales can be used to gain insights into the Turkish population. A one-dimensional structure was considered suitable for VAGUS-CR, whereas a three-dimensional structure was suitable for VAGUS-SR. We observed a correlation between VAGUS-CR and the 12th item of the PANSS general psychopathology subscale (G12) and SAI scale. Furthermore, VAGUS-SR was associated with BCIS, thereby providing insights via self-reports. In addition, VAGUS-SR and VAGUS-CR showed a moderate correlation. The level of insight evaluated with VAGUS-SR and VAGUS-CR increased with knowledge of schizophrenia. Upon investigating the relationship between perceived social support and insight, we identified a relationship between VAGUS-CR and only significant other subscales of MSPSS and between the VAGUS-SR D1 sub-dimension and significant other and total scores of MSPSS.

The SAI and PANSS G12 items administered by the clinician were used in the VAGUS-CR form for convergent validity analysis. Our findings are consistent with those of studies demonstrating a correlation between insight scales administered by clinicians [27, 50]. We observed a positive correlation between the two self-rated insight scales (BCIS and VAGUS-SR). This weak association may be attributed to the fact that BCIS measures a different insight structure. BCIS evaluates the patients’ thoughts about their experiences, perspectives on anomalous experiences, and self-confidence levels. In contrast, VAGUS-SR was developed to evaluate patients’ need for treatment, disease awareness, symptom attribution, and awareness of negative consequences [8]. Clinical insight is positively correlated with cognitive insight; however, some studies have not identified a relationship between them [7, 51–53], which is associated with different construct measurements of the scales [7]. Eventually, there was no relationship between the VAGUS-CR scale and BCIS, indicating that the person evaluating clinical insight (clinician or patient) may also affect the relationship between clinical and cognitive insight.

The PANSS G12 item displayed a strong and moderate correlation with the VAGUS-CR and VAGUS-SR total scores, suggesting that clinicians can assess insight with a single question from the PANSS scale to obtain a general idea of an individual’s insight. Evaluations made using the VAGUS scales will facilitate obtaining detailed information on this subject and will provide clues regarding areas where the clinician may need to work with the patient.

Insight can be evaluated differently between the clinicians and patients [12, 54]. When evaluating the relationship between different insight scales, a strong correlation was observed between the scales administered by clinicians [50]. In contrast, some studies found no association between the self-report scale and that performed by the clinician, while others found a low-to-moderate correlation [29, 55]. We identified a positive correlation between VAGUS-CR and VAGUS-SR, which is similar to the findings of Jeong et al. [27]. The moderate correlation between VAGUS-SR and VAGUS-CR supports the view that insight should be evaluated from different perspectives [29]. The data suggested that submitting the self-rated scales before clinically-rated scales increased the correlation between them; thus, submitting the VAGUS-SR scale before VAGUS-CR may have affected our findings [55].

In our study, we used CDSS for the discriminant validity of both VAGUS-CR and VAGUS-SR and found no relationship between them. Recent meta-analyses reported a weak positive relationship between insight/cognitive insight and depression in patients with schizophrenia [16, 51]; however, the relationship between different dimensions of insight and depression varies and is affected by methodological factors [16].

Regarding the single-factor structure of VAGUS-CR, Gerretsen et al. also identified one dimension for VAGUS-CR in a factor analysis, contrary to the theoretical four-factor model of insight [30]. This is compatible with the idea that the scales that assess insight and have different dimensions may comprise a single latent structure [30]. One effective factor may be retaining a limited number of questions in terms of the ease of using VAGUS-CR.

In our study, we determined three dimensions of factor analysis of the VAGUS-SR scale. The sub-dimensions are likely to have low internal consistency because they consist of few items. Gerretsen et al. obtained three factors in their factor analysis for VAGUS-SR, but the suggested four-factor structure was not confirmed [30]. In our study, the items included in the factors partially differed from those mentioned in previous results [5, 27, 30]. However, all three studies demonstrated that the “Symptom Attribution” dimension differs from others, which is similar to our data [5, 27, 30]. Two positively valenced items (Item 2: “My unusual or unique experiences are REAL regardless of what other people think about them.” and item 3: “The voices other people cannot hear are REAL regardless of what my doctor, family, or friends believe.”), which evaluate the “Symptom Attribution” sub-dimension, were separated from two negatively valenced items that were evaluated in the similar dimension. Moreover, they created a separate dimension in our study, similar to the results of Gerretsen et al. [30]. By contrast, de León et al. and Jeong et al. obtained a similar factor with a different item added to these two items (2,3, and 8 vs. 2,3, and 9, respectively) [5, 27]. Jeong et al. suggested that the VAGUS-SR scale “Symptom Attribution” factor may be a component separate from clinical insight [27]. Considering that cognitive insight deals with the patient’s evaluation of their perspectives on thoughts and comments, and accepting the possibility of being incorrect, Jeong et al. considered that the “Symptom Attribution” factor may have been affected by cognitive insight owing to the item content. In addition, the authors recommended a study using BCIS. We did not identify a relationship between the “Symptom Attribution” factor (items 2 and 3) and cognitive insight (BCIS) or self-reflectiveness sub-dimension of BCIS, which includes the willingness to acknowledge fallibility. Our inability to identify a relationship between BCIS and the D2 sub-dimensions of VAGUS-SR may be attributed to the fact that BCIS evaluates different areas. Our findings revealed that inquiring patients about the cause of their psychotic experiences in different ways provided diverse information. Although there was no relationship between BCIS and the D2 dimensions, this supported the theory of Beck et al. regarding the difference between clinical and emotional insights [8]. The relationship between the BCIS self-reflectiveness subscale and VAGUS-SR D1 sub-dimension was partially identical to that observed in the study by Beck et al. [8]. In light of our findings, there could be certain relationships between the D2 sub-dimension, which revealed that the individual displayed an awareness of thinking differently from other people, and certain sub-dimensions of the Insight Scale (particularly with the sub-dimension containing awareness items related to disturbed thinking) developed by Markova et al. [56]. The Insight Scale focuses on evaluating the awareness of the changes experienced by patients rather than their beliefs about these changes. There may be a relationship among sub-dimensions containing the items on awareness, thus necessitating additional research [56].

Studies have shown that psychoeducation in addition to pharmacotherapy and psychological interventions, including cognitive-behavioral therapy, metacognitive therapy, metacognitive reflection insight therapy, and metacognitive training, improves insight in patients with schizophrenia [57]. In our study, the level of insight evaluated using VAGUS-SR and VAGUS-CR increased with knowledge of schizophrenia. Some studies reported an association between increased knowledge of the disease and greater insight [19, 20]. A recent study used movies to present information regarding schizophrenia and demonstrated increased insight following the training [21]. These findings highlight the importance of psychoeducational studies in a psychotic patient group [42]. However, some researchers have demonstrated that there is no change in insight with psychoeducation [22–24]. Nonetheless, education has increased knowledge regarding schizophrenia [24]. Researchers have identified the following possible factors that may be effective in this situation: the inability to use novel semantic information appropriately in patients with schizophrenia and clinician- or self-rated differences in the scales used to evaluate knowledge and insight about schizophrenia.

Upon investigating the relationship between perceived social support and insight, we identified a positive relationship between VAGUS-CR and MSPSS significant other subscales and between the VAGUS-SR scale sub-dimension D1 and MSPSS significant other and MSPSS total scores. Few studies have investigated the relationship between social support and insight in schizophrenia. However, more than half of the patients with schizophrenia experience poor social support [14]. Kaiser et al. (2006) mentioned that social support comprises two dimensions (network size and satisfaction with support); they emphasized that satisfaction with support may be more important for individuals with serious mental illnesses [25]. Goldberg et al. did not detect a relationship between network size and insight, thus supporting this assumption [58]. Kaiser et al. (2006) could not identify a significant linear correlation between insight and satisfaction with support. However, upon reanalyzing their data considering the possibility of curvilinear relationships, individuals with moderate insight experienced less satisfaction with social support (satisfaction with support) than those with low and high levels of insight [25]. In another study investigating the relationship between social interaction and insight, the researchers considered social contact frequency, number of contacts, and satisfaction. The investigators found that insight was positively correlated with frequent contact with friends and family and negatively correlated with contact satisfaction with friends [26]. The group with the lowest level of insight exhibited the highest level of social isolation. Patients with better insight may be less satisfied with their peer relationships because of higher expectations and the need for support from their friends. However, a recent study with a large sample size did not demonstrate a relationship between social support and insight [14]. The use of a more detailed scale to evaluate social support in our study may have generated different results. Unlike other studies, we used self-rated scales to evaluate these variables, which may have been more effective in detecting the relationship between perceived social support and insight. Considering the negative relationship between social support and depression and data on the relationship between insight and depression, researchers should perform detailed studies to better evaluate the relationship between social support and insight [16, 25].

### Limitations

Considering that the studies demonstrated an unstable structure of insight, the first limitation was that we could only evaluate correlations owing to the cross-sectional research design and limited number of patients. Second, seeking clinical stability as the minimum criteria for patients to submit the scales, resulted in limited validity of our findings. Third, the necessity of working only on the scales of individuals with auditory hallucinations and the most prominent delusions is a limitation of the factor analysis of the VAGUS scales. During the development of the scale, evaluation of auditory hallucinations and specific delusions by both versions of the VAGUS scale was considered a limitation [30]. Fourth, we did not perform test-retest reliability in this study. In addition, the scales only evaluate the present symptoms, and no information can be obtained regarding insight in the past stages of the disease. Since our study was conducted in individuals over the age of 18 years, there may be differences in the validity of the scales and the results obtained in adolescent studies. Although it was close to 0.70 in our study, the poor Cronbach’s alpha values of the Turkish version of the Beck Cognitive Insight Scale and the lack of validity and reliability of the Turkish version of CGI-S were among our limitations and should be considered when evaluating our results. Despite its widespread use, validity and reliability studies on CGI are lacking in many languages. Finally, our study had a high dropout rate. Research on the effectiveness of different strategies for participation in clinical trials is ongoing, and it has been stated that these strategies may change depending on the study design and study population, and that more studies are needed in this area [59]. Invitations to participate in this study at the CMHC were made by case counselors with strong therapeutic relationships with patients, which increased their willingness to participate. As the patients included in the study were clinically stable, they usually underwent drug treatments and preferred not to stay in the clinic for a long time. Therefore, invitations to these individuals were made in the waiting rooms. In addition, arrangements were made for individuals who wished to participate in the study for follow-up appointments. However, some patients were reluctant to see a clinician other than their case counselor unless they believed that they had signs of illness. In addition, although some individuals were more comfortable completing self-report scales, they wanted to avoid meeting the clinicians. In contrast, others were more positive about the interview but were reluctant to complete the scales.

## Conclusions

In summary, insight is a concept defined by different dimensions in which research is ongoing, and several tools are used for its evaluation. Considering the differences between the findings obtained using these tools and the multidimensional effects of insight in patients with schizophrenia, the use of scales that allow for a detailed assessment and evaluation of the insight of individuals from different perspectives will be useful. We determined the reliability and validity of VAGUS-SR and VAGUS-CR in the Turkish population. In addition, the positive relationship between perceived social support and insight emphasizes the importance of increasing social support through interventions aimed at improving insight.

## Supporting information

S1 Table Distribution of the scales. (DOCX)

S2 Table Estimated values and threshold values of fit indices for the VAGUS-CR form. (DOCX)

S3 Table Estimated values and threshold values of fit indices for VAGUS-SR form. (DOCX)

S4 Table Estimated reliability values of other measurement tools. (DOCX)

S5 Table Descriptive statistics on measurement tools. (DOCX)

S1 File This is the study’s underlying data set. (SAV)
