# Supplementary material for: Insight in schizophrenia is associated with psychoeducation and social support: Testing a new more comprehensive insight tool in Turkish schizophrenia patients
Source: PLoS One. 2023 Jul 7;18(7):e0288177. doi: 10.1371/journal.pone.0288177 (PMC10328252; doi:10.1371/journal.pone.0288177)
Supplement: S1 Table — (DOCX) [file pone.0288177.s001.docx]

**S1 Table. Distribution of the scales**

|  | Kolmogorov-Smirnov | | | Shapiro-Wilk | | |
| --- | --- | --- | --- | --- | --- | --- |
|  |  | sd | *p* |  | sd | *p* |
| VAGUS-CR | .221 | 58 | .000 | .875 | 58 | .000 |
| VAGUS-SR | .110 | 58 | .078* | .965 | 58 | .093* |
| SAI | .104 | 58 | .181* | .927 | 58 | .002 |
| KASQ | .113 | 58 | .063* | .972 | 58 | .196* |
| BCIS | .112 | 58 | .068* | .976 | 58 | .304* |
| MSPSS | .102 | 58 | .200* | .955 | 58 | .033 |
| PANSS | .096 | 58 | .200* | .955 | 58 | .033 |
| CDSS | .167 | 58 | .000 | .908 | 58 | .000 |

*p ˃ .05. SAI, Schedule for the Assessment of Insight; KASQ, Knowledge About Schizophrenia Questionnaire; BCIS, Beck Cognitive Insight Scale; MSPSS, Multidimensional Scale of Perceived Social Support (MSPSS); PANNS, Positive and Negative Syndrome Scale; CDSS, Calgary Depression Scale for Schizophrenia
