# Supplementary material for: Insight in schizophrenia is associated with psychoeducation and social support: Testing a new more comprehensive insight tool in Turkish schizophrenia patients
Source: PLoS One. 2023 Jul 7;18(7):e0288177. doi: 10.1371/journal.pone.0288177 (PMC10328252; doi:10.1371/journal.pone.0288177)
Supplement: S2 Table — (DOCX) [file pone.0288177.s002.docx]

**S2 table. Estimated values and threshold values of fit indices for the VAGUS-CR form**

| Fit Indices | Good Fit | Acceptable Fit | Estimated Values |
| --- | --- | --- | --- |
| S-Bχ2 /sd | 0≤ χ2/sd <2 | 2 ≤χ2/sd ≤5 | 7.07/5 = 1.41 |
| RMSEA(%90GA) | 0≤RMSEA<0.05 | 0.05≤RMSEA≤0.10 | 0.092 (0.000-0.230) |
| NNFI | 0.95≤NNFI≤1.00 | 0.90 ≤NNFI< 0.95 | 0.93 |
| NFI | 0.95≤NFI≤1.00 | 0.90 ≤NFI< 0.95 | 0.90 |
| CFI | 0.95 ≤CFI≤ 1.00 | 0.90 ≤CFI< 0.95 | 0.96 |
| GFI | 0.95 ≤GFI≤ 1.00 | 0.90 ≤GFI< 0.95 | 0.94 |
| AGFI | 0.95 ≤AGFI≤ 1.00 | 0.90 ≤AGFI< 0.95 | 0.82 |
| SRMR | 0≤ SRMR <0.05 | 0.05≤ SRMR ≤0.10 | 0.13 |
