# Supplementary material for: Insight in schizophrenia is associated with psychoeducation and social support: Testing a new more comprehensive insight tool in Turkish schizophrenia patients
Source: PLoS One. 2023 Jul 7;18(7):e0288177. doi: 10.1371/journal.pone.0288177 (PMC10328252; doi:10.1371/journal.pone.0288177)
Supplement: S4 Table — (DOCX) [file pone.0288177.s004.docx]

**S4 table. Estimated reliability values of other measurement tools**

| Scales and sub-dimensions | Cronbach α | McDonald ω | Stratified Cronbach α |
| --- | --- | --- | --- |
| BCIS_self-reflectiveness | .668 | .693 | -- |
| BCIS_self-certainty | .695 | .699 | -- |
| BCIS_composite_index | -- | -- | .621 |
| MSPSS - Family | .882 | .888 | -- |
| MSPSS - Friends | .911 | .912 | -- |
| MSPSS - Significant other | .887 | .888 | -- |
| MSPSS - Family | -- | -- | .969 |
| KASQ | .765 | .771 | -- |
| SAI | .749 | .813 | -- |

SAI, Schedule for the Assessment of Insight; KASQ, Knowledge About Schizophrenia Questionnaire; BCIS, Beck Cognitive Insight Scale; MSPSS, Multidimensional Scale of Perceived Social Support (MSPSS)
