# Supplementary material for: Insight in schizophrenia is associated with psychoeducation and social support: Testing a new more comprehensive insight tool in Turkish schizophrenia patients
Source: PLoS One. 2023 Jul 7;18(7):e0288177. doi: 10.1371/journal.pone.0288177 (PMC10328252; doi:10.1371/journal.pone.0288177)
Supplement: S5 Table — (DOCX) [file pone.0288177.s005.docx]

**S5 table. Descriptive statistics on measurement tools**

|  | Min-Max | $\bar{X}$ | SD | Skewness | Kurtosis |
| --- | --- | --- | --- | --- | --- |
| VAGUS_CR | 0.00-50.00 | 33.06 | 14.33 | -.92 | -.33 |
| VAGUS_SR-D1 | 0.00-50.00 | 30.99 | 14.14 | -.48 | -.78 |
| VAGUS_SR-D2 | -0.17-20.00 | 7.97 | 6.05 | -.10 | .37 |
| VAGUS_SR-D3 | 0.00-20.00 | 8.64 | 5.89 | .30 | -.79 |
| VAGUS_SR | 0.00-90.00 | 50.58 | 19.65 | -.44 | -.42 |
| SAI | 0.00-20.00 | 13.91 | 4.85 | -.82 | .37 |
| KASQ | 5.00-21.00 | 12.57 | 3.91 | -.07 | -.72 |
| MSPSS - Family | 4.00-28.00 | 22.14 | 6.20 | -1.16 | .92 |
| MSPSS - Friends | 4.00-28.00 | 16.15 | 8.29 | .03 | -1.33 |
| MSPSS - Significant other | 4.00-28.00 | 16.60 | 8.79 | -.04 | -1.42 |
| MSPSS Total | 12.00-84.00 | 54.89 | 18.45 | -.05 | -.51 |
| PANSS- Positive | 7.00-41.00 | 11.28 | 5.81 | 2.82 | 11.34 |
| PANSS- Negative | 7.00-26.00 | 14.71 | 5.57 | .12 | -1.15 |
| PANSS- General psychopathology | 17.00-46.00 | 27.21 | 7.40 | .61 | -.51 |
| PANNS Total | 31.00-104.00 | 53.36 | 15.29 | .65 | .62 |
| CDSS | 0.00-13.00 | 3.74 | 3.29 | .71 | -.29 |
| BCIS_self-reflectiveness | 12.00-36.00 | 22.58 | 5.16 | .17 | -.20 |
| BCIS_self-certainty | 6.00-23.00 | 14.62 | 3.86 | .28 | .09 |
| BCIS_composite_index | -7.00-21.00 | 7.97 | 5.95 | -.10 | .37 |

SAI, Schedule for the Assessment of Insight; KASQ, Knowledge About Schizophrenia Questionnaire; BCIS, Beck Cognitive Insight Scale; MSPSS, Multidimensional Scale of Perceived Social Support (MSPSS); PANNS, Positive and Negative Syndrome Scale; CDSS, Calgary Depression Scale for Schizophrenia; CGI, Clinical Global Impressions
